# Cognitive Impairment as a Predictor of Long-Term Outcomes in Septic Patients: A Retrospective Observational Study

**DOI:** 10.7759/cureus.90495

**Published:** 2025-08-19

**Authors:** Hiroyuki Koami, Yuichiro Sakamoto, Yutaro Furukawa, Kosuke Mouri, Ayaka Matsuoka, Kota Shinada, Kento Nakayama, Sachiko Iwanaga, Shogo Narumi, Mayuko Koba

**Affiliations:** 1 Department of Emergency and Critical Care Medicine, Saga University, Saga, JPN

**Keywords:** cognitive impairment, long-term outcome, retrospective study, sensitivity analysis, sepsis

## Abstract

Background

The importance of long-term outcomes after sepsis has been increasingly emphasized in recent years; however, little is known about the relationship between pre-sepsis patient characteristics and long-term outcomes. This study examined the impact of patient characteristics pre-admission, specifically physical, cognitive, and emotional disorders, on long-term clinical outcomes of sepsis.

Methods

This was a single-center, retrospective study. From August 2014 to July 2016, this study included adult patients admitted to our hospital with a diagnosis of sepsis who were followed up for one year. These 76 patients were classified as Survivors (n=31; 40.8%) or Deceased (n=45; 59.2%), based on their one-year outcomes.

Results

In this study, sepsis patients (n=45; 59.0%) died within one year. Univariate analysis demonstrated that several factors were associated with 1-year mortality, including male, arrhythmias, Sepsis-2 severity, activities of daily living (ADL) difficulties, and cognitive impairment. Multiple logistic regression models employing significant parameters from univariate analysis revealed that cognitive impairment (CI) is an independent risk factor for 1-year outcome (OR 5.427, 95% CI 1.385-21.260, P=0.0152). Patient history and needs assessment revealed that all patients with pre-admission cognitive impairment were discharged bedridden after sepsis treatment, and they exhibited a significantly lower survival rate (p=0.0332) compared to patients without cognitive impairment.

Conclusion

These results indicate that pre-septic cognitive impairment is a significant independent predictor of poor long-term clinical outcomes for sepsis patients.

## Introduction

Sepsis is a pressing societal issue with a global impact [1]. The Surviving Sepsis Campaign Guidelines (SSCG), launched in 2004, have significantly enhanced awareness of sepsis and have improved the quality of sepsis care [2]. Recent estimates suggest that 31.5 million cases of sepsis occur globally each year, including 19.4 million cases of severe sepsis and 5.3 million possible deaths, primarily in high-income countries [1].

Although advances in sepsis treatment have increased survival rates, sepsis remains a significant public health issue [3, 4]. A substantial number of sepsis survivors face a poor long-term prognosis, with observational studies reporting 1-year mortality rates ranging from 21.5-71.9% [5, 6]. Moreover, deaths from sepsis continue to be reported even several years after hospital discharge, suggesting that using 28-day mortality as a primary endpoint in clinical studies may be suboptimal [6, 7].

The long-term prognosis for sepsis survivors is challenging, characterized not only by high mortality rates but also by persistent disabilities such as physical, cognitive, and mental impairments [8]. Particularly, a significant number of patients, especially the elderly, experience new or worsening cognitive impairment, which has been shown to increase during hospitalization and is associated with a shorter lifespan [9-13]. These post-septic impairments often severely impact independent daily living and can lead to poor outcomes [10].

Despite a wealth of data on post-septic cognitive impairment and its association with clinical outcomes, there are few reports regarding the correlation between pre-septic cognitive impairment and long-term outcomes. In this study, we retrospectively investigated the relationship between pre-hospitalization patient characteristics (including cognitive, physical, and emotional disorders) and long-term clinical outcomes of sepsis.

## Materials and methods

Study design and patients

This retrospective observational study was conducted at Saga University Hospital in Japan. Patients aged 18 years or older who were admitted to the hospital with sepsis between August 2014 and July 2016, and whose one-year outcome was confirmed, were included in this study. Patients who developed sepsis after hospital admission or whose one-year outcome could not be confirmed were excluded. All cases were categorized into two groups based on their one-year outcome: Survivors (n=31; 40.8%) and Deceased (n=45; 59.2%).

Variables related to patient characteristics and medical history

Patient data, including age, percentage of patients over 65 years old, sex, prior hospitalizations, transfer cases, and past medical problems were recorded, including conditions such as hypertension, arrhythmia, cardiovascular disease, peripheral vascular disease, cerebrovascular disease, diabetes mellitus, gastrointestinal and hepatobiliary disease, hyperlipidemia, malignancy, chronic respiratory disease, and chronic kidney disease.

Clinical scores and blood sampling

We calculated several clinical scores as follows: the systemic inflammatory response syndrome (SIRS) score [14], the Japanese Association for Acute Medicine (JAAM) disseminated intravascular coagulation (DIC) score [15], the acute physiology and chronic health evaluation (APACHE) II score, the sequential organ failure assessment (SOFA) score, and the quick SOFA (qSOFA) score [16]. SOFA scores were computed for each organ component, in addition to the total score. Organ failure was further defined as a score of 2 or more for a given organ. Evaluations were also conducted on the number of organ failures and on serum lactate levels. These clinical severities were mainly assessed on the day of admission.

Sepsis management at our hospital and clinical outcomes

During the study period, sepsis was diagnosed and treated based on the Sepsis-2 definition based on the presence of infection and at least two of the following: temperature >38°C or <36°C, heart rate >90/min, respiratory rate >20/min or partial pressure of carbon dioxide (PaCO₂) <32 mmHg, WBC >12,000/µL, <4,000/µL, or >10% immature forms [17]. Therefore, to compare data using the current definition of Sepsis-3, defined as an acute increase in SOFA score ≥2 points consequent to infection [16], we also assessed sepsis occurrence according to Sepsis-3 criteria.

Additionally, data were collected on infection sources, pathogenic bacteria, and results of blood cultures. Sepsis treatments were performed by emergency physicians and critical care physicians at the facility. However, there is no institutional protocol for managing sepsis at this hospital. This study documents various treatments administered during each hospital stay, including antibiotics, antifungal and antiviral agents, mechanical ventilation, renal replacement therapy, anti-DIC therapy, catecholamines, steroids, insulin use, transfusions, and surgical interventions.

Indicators for comprehensive functional assessment

Upon admission to and discharge from Saga University Hospital, patients or their relatives are interviewed by nurses for a comprehensive functional assessment. This pre-admission assessment comprises various indices, such as “indicators on activity of daily living and cognitive-emotional functions”, as well as “extent of being bedridden (“Netakiri” index)”. The former encompasses basic difficulties with activities of daily living (ADL), such as required assistance with toileting and moving about the residence, as well as instrumental ADL difficulties, such as an inability to leave the ward for examinations or to go to the store alone. Other factors contributing to the condition include lethargy, cognitive impairment such as memory problems, an inability to engage in simple conversations, and emotional disturbances such as depression.

The Netakiri index classifies independence into four categories: “Rank J (independent in daily life)”, “Rank A (independent indoors, but needs support when going out)”, “Rank B (needs support even indoors)”, and “Rank C (in bed all day)”. The Barthel Index was also utilized to measure post-discharge indicators. This index consists of 10 domains, such as feeding, wheelchair-to-bed mobility, personal toilet, getting on and off the toilet, self-bathing, walking on level surfaces, stair-climbing, dressing, and bowel and bladder control.

Variables in clinical outcomes

In this study, we collected clinical outcome data, including lengths of hospital stays, transfers to other hospitals, hospital mortality, and one-year survival rates. The outcome after one year was confirmed by calling the hospital or nursing home to which the patient was transferred. The composite outcome of death or tendency to be bedridden (Rank B or C of Netakiri index) at discharge was defined and evaluated for correlation with the presence or absence of cognitive impairment.

Statistical analysis

Data access for this study was initiated on February 6, 2018. Only HK, the person in charge of data analysis, had access to information that could identify individual participants after data collection. After anonymization, other authors also used these data. To evaluate factors associated with long-term outcomes, patients were categorized based on death within a year and were subjected to univariate analysis. The median (quartile (Q)1, Q3) was used to represent continuous variables, whereas numbers (percentages) were employed for categorical/nominal variables in both groups.

The Mann-Whitney U test was utilized for continuous variables, whereas the Chi-square test and Fisher’s exact test were applied to categorical variables. The Z-value was calculated using the Wilcoxon test, and the chi-square value was calculated using the chi-square test. On the other hand, Fisher's exact test does not have conventional test statistics, so it was marked as N/A. Statistical significance was defined as p < 0.05. In this study, we did not perform any corrections for multiple testing; therefore, all results should be considered exploratory and should be interpreted cautiously. Logistic regression analysis was conducted to identify predictors of one-year survival, utilizing cognitive impairment as well as factors that showed significant differences in univariate analysis. We conducted a sensitivity analysis only for patients diagnosed with Sepsis-3 definition (n = 53; 69.7%).

Finally, we constructed Kaplan-Meier curves to analyze the presence or absence of cognitive impairment and conducted log-rank tests. All statistical analyses were performed utilizing JMP® Pro 16.1.0 software (SAS Institute, Cary, NC, USA).

## Results

**Figure 1:**
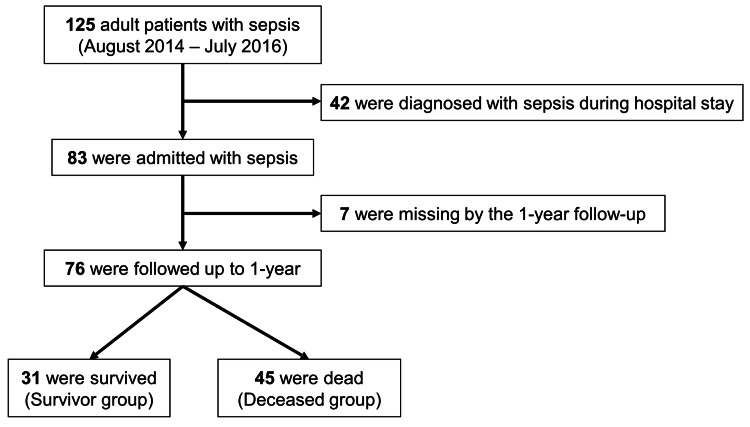
Flow diagram of the present study. During the study, 125 adult patients with sepsis were identified. After excluding 42 patients who were diagnosed after admission, as well as 7 patients whose 1-year outcome was unknown, 76 patients (31 survivors (40.8%) and 45 deceased (59.2%)) were enrolled.

During the two-year observational period, our hospital diagnosed 125 patients with sepsis (Fig. 1). Of these patients, 83 were initially hospitalized for sepsis, and 42 were diagnosed with sepsis after being hospitalized for another medical condition. After excluding seven patients whose 1-year outcomes were unknown, the present study enrolled 76 patients. All participants were categorized as survivors (N=31; 40.8%) or deceased (N=45; 59.2%) 1 year after discharge.

Table 1 shows that factors strongly associated with one-year mortality from sepsis include male gender, history of arrhythmia, and organ dysfunction scores such as the SOFA score. Survivors included significantly fewer males (Survivors: 11 (35.5%) vs. Deceased: 36 (80.0%), P<0.0001); however, there were no significant differences with regard to age, percentage of older patients, history of hospitalization within 1 year, or transfers to other institutions. The percentage with a history of arrhythmia was significantly lower among survivors than deceased patients in terms of past medical history (1 (3.2 %) vs. 12 (26.7 %), P=0.01). Other medical conditions, however, revealed no statistically significant differences between the two groups. While some clinical scores, including SIRS, JAAM DIC, and qSOFA, were the same in both groups, median values of the APACHE II score (18 (16, 22) vs. 22 (18, 30), P=0.02) and the total SOFA score (5 (3, 8) vs. 8 (6, 11), P<0.0001) were considerably lower among survivors than among deceased patients. SOFA scores for each organ of survivors were significantly lower in the central nervous system (CNS)(1 (0, 1) vs. 2 (1, 3), P=0.002), respiratory system (1 (0, 1) vs. 2 (1, 3), P=0.01), and liver (0 (0, 0) vs. 0 (0, 1), P=0.04), respectively. Additionally, the proportion of failure for each organ was significantly lower in the CNS (5 (16.1%) vs. 24 (53.3%), P=0.0016), respiratory system (7 (22.6%) vs. 23 (51.1%), P=0.01), and coagulation system (6 (19.4%) vs. 23 (51.1%), P=0.005) of survivors compared to deceased patients. Consequently, the incidence of organ failure was significantly lower among survivors (1 (1, 2) vs. 3 (2, 4), P<0.0001).

**Table 1:** Patient characteristics and clinical scores of survivors and deceased patients. The median (Q1, Q3) was used to represent continuous variables, whereas number (%) was used for categorical/nominal variables in both groups. The Mann-Whitney U test was utilized for continuous variables, whereas Chi-square and Fisher’s exact tests were applied for categorical variables. The Z-value (†) was calculated using the Wilcoxon test, and the chi-square value (‡) was calculated using the chi-square test. On the other hand, Fisher's exact test does not have conventional test statistics, so it was marked as N/A(*). Statistical significance in the Mann-Whitney U test, Chi-square, and Fisher’s exact tests was defined as p< 0.05. SIRS: systemic inflammatory response syndrome; JAAM: Japanese association for acute medicine; DIC: disseminated intravascular coagulation; qSOFA: quick sequential organ failure assessment; APACHE: acute physiology and chronic health evaluation; CNS: central nervous system.

		Survivors (n=31)	Deceased (n=45)	Test statistic	P value (< 0.05)
Age, y/o, median (Q1, Q3)		77 (57, 84)	74 (66, 81)	-0.122^†^	0.90
Age>65, n (%)		20 (64.5)	36 (80.0)	2.269^‡^	0.13
Male, n (%)		11 (35.5)	36 (80.0)	15.414^‡^	<0.0001
Hospitalization within 1 year, n (%)		11 (35.5)	17 (37.8)	0.042^‡^	0.84
Transfer cases, n (%)		17 (54.8)	25 (55.6)	0.004^‡^	0.95
Past medical history	Hypertension, n (%)	16 (51.6)	21 (46.7)	0.180^‡^	0.67
	Arrhythmia, n (%)	1 (3.2)	12 (26.7)	N/A^*^	0.01
	Cardiovascular disease, n (%)	7 (22.6)	14 (31.1)	0.668^‡^	0.41
	Peripheral vascular disease, n (%)	2 (6.5)	5 (11.1)	N/A^*^	0.69
	Cerebrovascular disease, n (%)	9 (29.0)	12 (26.7)	0.051^‡^	0.82
	Diabetes mellitus, n (%)	9 (29.0)	13 (28.9)	0.000^‡^	0.99
	Gastrointestinal and hepatobiliary disease, n (%)	10 (32.3)	11 (24.4)	0.560^‡^	0.45
	Hyperlipidemia, n (%)	6 (19.4)	5 (11.1)	N/A^*^	0.34
	Malignancy, n (%)	7 (22.6)	14 (31.1)	0.668^‡^	0.41
	Chronic respiratory disease, n (%)	4 (12.9)	7 (15.6)	N/A^*^	1.00
	Chronic kidney disease, n (%)	6 (19.4)	7 (15.6)	0.187^‡^	0.67
SIRS score, median (Q1, Q3)		3 (2, 4)	3 (2, 3)	1.724^†^	0.08
JAAM DIC score, median (Q1, Q3)		4 (2, 6)	4 (2, 7)	-0.740^†^	0.46
qSOFA score, median (Q1, Q3)		2 (1, 3)	2 (1, 3)	-0.629^†^	0.53
APACHEII score, median (Q1, Q3)		18 (16, 22)	22 (18, 30)	-2.269^†^	0.02
SOFA score	Total, median (Q1, Q3)	5 (3, 8)	8 (6, 11)	-4.261^†^	<0.0001
	CNS, median (Q1, Q3)	1 (0, 1)	2 (1, 3)	-3.184^†^	0.002
	CNS ≥ 2, n (%)	5 (16.1)	24 (53.3)	10.767^‡^	0.001
	Respiration, median (Q1, Q3)	1 (0, 1)	2 (1, 3)	-2.568^†^	0.01
	Respiration ≥ 2, n (%)	7 (22.6)	23 (51.1)	6.254^‡^	0.01
	Cardiovascular, median (Q1, Q3)	0 (0, 3)	0 (0, 3)	-1.382^†^	0.17
	Cardiovascular ≥ 2, n (%)	9 (29.0)	19 (42.2)	1.372^‡^	0.24
	Renal, median (Q1, Q3)	1 (0, 2)	2 (1, 2)	-0.947^†^	0.34
	Renal ≥ 2, n (%)	12 (38.7)	24 (53.3)	1.574^‡^	0.21
	Liver, median (Q1, Q3)	0 (0, 0)	0 (0, 1)	-2.076^†^	0.04
	Liver ≥ 2, n (%)	4 (12.9)	10 (22.2)	1.061^‡^	0.30
	Coagulation, median (Q1, Q3)	0 (0, 1)	2 (0, 2)	-1.723^†^	0.08
	Coagulation ≥ 2, n (%)	6 (19.4)	23 (51.1)	7.844^‡^	0.005
No of Organ Failure, median (Q1, Q3)		1 (1, 2)	3 (2, 4)	-4.466^†^	<0.0001
Lactate, mmol/L, median (Q1, Q3)		3.1 (2.2, 5.0)	5.1 (2.0, 7.1)	-1.708^†^	0.09

Table 2 shows a significant relationship between one-year outcomes for sepsis and the severity of sepsis based on the Sepsis-2 definition. In regard to Sepsis-2, survivors had significantly fewer severe cases, such as septic shock (p=0.02). Of 76 septic patients with Sepsis-2, 53 (69.7%) patients were diagnosed based on the Sepsis-3 definition, with 20 (37.7%) survivors and 33 (62.3%) deceased patients diagnosed with sepsis and septic shock. A similar trend was observed using the Sepsis-3-based classification; however, no significant difference was found. Furthermore, there was statistical equality among sources of infection, pathogenic bacteria, and the number of positive blood cultures.

**Table 2:** Indicators on infections and sepsis. Number (%) was used as categorical/nominal variables in both groups. Chi-square and Fisher’s exact tests were applied for categorical variables. The Chi-square value (‡) was calculated using the Chi-square test. On the other hand, Fisher's exact test does not have conventional test statistics, so it was marked as N/A(*). Statistical significance in the Chi-square and Fisher’s exact tests was defined as p< 0.05.

		Survivors (n=31)	Deceased (n=45)	Test statistic	P value (< 0.05)
Sepsis-2 definition	Sepsis, n (%)	11 (35.5)	5 (11.1)	7.446^‡^	0.02
	Severe sepsis, n (%)	8 (25.8)	11 (24.4)		
	Septic shock, n (%)	12 (38.7)	29 (64.4)		
Sepsis-3 definition	Sepsis, n (%)	8/20 (40.0)	9/33 (27.3)	0.926^‡^	0.34
	Septic shock, n (%)	12/20 (60.0)	24/33 (72.7)		
Infection sources	Respiratory, n (%)	2 (6.5)	13 (28.9)	7.743^‡^	0.10
	Urinary, n (%)	10 (32.3)	7 (15.6)		
	Abdominal, n (%)	9 (29.0)	10 (22.2)		
	Soft tissue, n (%)	4 (12.9)	8 (17.8)		
	Others, n (%)	6 (19.4)	7 (15.6)		
Pathogenic bacteria	Gram-positive, n (%)	13 (41.9)	21 (46.7)	2.552^‡^	0.47
	Gram-negative, n (%)	12 (38.7)	17 (37.8)		
	Mixed-type, n (%)	1 (3.2)	4 (8.9)		
	Not detected, n (%)	5 (16.1)	3 (6.7)		
Positive blood culture, n (%)		4 (15.4)	9 (21.4)	N/A^*^	0.75

Table 3 shows that ADL difficulties and cognitive impairment pre-admission hospitalization were significantly associated with one-year mortality due to sepsis. Survivors demonstrated better functional and cognitive status prior to sepsis compared to non-survivors. Specifically, survivors had significantly lower rates of ADL difficulties (8 (25.8%) vs. 22 (48.9%), p=0.04) and cognitive impairment (7 (22.6%) vs. 21 (46.7%), P=0.03) compared to patients who later deceased. The two groups were statistically similar in terms of instrumental ADL (IADL) difficulties, decreased motivation, emotional disturbance, and Netakiri index.

**Table 3:** Comprehensive functional assessment indices before admission. Number (%) was used for categorical/nominal variables in both groups. Chi-square and Fisher’s exact tests were applied for categorical variables. The Chi-square value (‡) was calculated using the Chi-square test. On the other hand, Fisher's exact test does not have conventional test statistics, so it was marked as N/A(*). Statistical significance in the Chi-square and Fisher’s exact tests was defined as p< 0.05. ADL: activities of daily living; IADL: instrumental activities of daily living.

		Survivors (n=31)	Deceased (n=45)	Test statistic	P value (< 0.05)
Pre-admission indicators on the ADL and cognitive-emotional functions	ADL difficulties, n (%)	8 (25.8)	22 (48.9)	4.093^‡^	0.04
	IADL difficulties, n (%)	20 (64.5)	33 (73.3)	0.676^‡^	0.41
	Decreased motivation, n (%)	0 (0.0)	4 (8.9)	N/A^*^	0.14
	Cognitive impairment, n (%)	7 (22.6)	21 (46.7)	4.576^‡^	0.03
	Emotional disturbance, n (%)	1 (3.2)	2 (4.4)	N/A^*^	1.00
Pre-admission Levels of bed-ridden degree (“Netakiri” index)	Rank J, n (%)	10 (32.3)	11 (24.4)	5.593^‡^	0.13
	Rank A, n (%)	14 (45.2)	13 (28.9)		
	Rank B, n (%)	5 (16.1)	10 (22.2)		
	Rank C, n (%)	2 (6.5)	11 (24.4)		

According to Table 4, the use of catecholamines and steroids was strongly associated with mortality one year after sepsis. Survivors had significantly lower rates of treatment with catecholamines (16 (51.6%) vs. 38 (84.4%), p=0.002) and steroids (8 (25.8%) vs. 23 (51.1%), p=0.03) than patients who later deceased. No significant differences were found in other treatments irrespective of 1-year outcome. Survivors showed significant improvements in clinical outcomes compared to the deceased group, with longer hospital stays (28 (15, 69) days vs. 12 (3, 24) days, p=0.0006), lower hospital mortality (0 (0.0%) vs. 28 (62.2%), P<0.0001), and longer survival time within one year (365 (365, 365) days vs. 15 (3, 77) days, p<0.0001).

**Table 4:** Treatments and clinical outcomes. Median (Q1, Q3) was used to represent continuous variables, whereas number (%) was used for categorical/nominal variables in both groups. The Mann-Whitney U test was utilized for continuous variables, whereas Chi-square and Fisher’s exact tests were applied for categorical variables. The Z-value (†) was calculated using the Wilcoxon test, and the Chi-square value (‡) was calculated using the Chi-square test. On the other hand, Fisher's exact test does not have conventional test statistics, so it was marked as N/A(*). Statistical significance in the Mann-Whitney U test, Chi-square, and Fisher’s exact tests was defined as p< 0.05. DIC: disseminated intravascular coagulation.

	Survivors (n=31)	Deceased (n=45)	Test statistic	P value (< 0.05)
Antibiotics, n (%)	31 (100.0)	45 (100.0)	---	---
Antifungal agents, n (%)	3 (9.7)	6 (13.3)	N/A^*^	0.73
Antiviral agents, n (%)	1 (3.2)	1 (2.2)	N/A^*^	1.00
Mechanical ventilation, n (%)	11 (35.5)	26 (57.8)	3.652^‡^	0.06
Renal replacement therapy, n (%)	15 (48.4)	16 (35.6)	1.251^‡^	0.26
Anti-DIC agents, n (%)	18 (58.1)	26 (57.8)	0.001^‡^	0.98
Catecholamines, n (%)	16 (51.6)	38 (84.4)	9.620^‡^	0.002
Steroids, n (%)	8 (25.8)	23 (51.1)	4.866^‡^	0.03
Insulin use, n (%)	8 (25.8)	11 (24.4)	0.018^‡^	0.89
Transfusions, n (%)	5 (16.1)	12 (26.7)	1.174^‡^	0.28
Surgical interventions, n (%)	12 (38.7)	13 (28.9)	0.802^‡^	0.37
Length of hospital stay, days, median (Q1, Q3)	28 (15, 69)	12 (3, 24)	3.444^†^	0.0006
Hospital mortality, n (%)	0 (0.0)	28 (62.2)	N/A^*^	<0.0001
Transferred to the other hospital, n (%)	19 (65.5)	14 (82.4)	N/A^*^	0.22
Survival days within 1 year, days, median (Q1, Q3)	365 (365, 365)	15 (3, 77)	7.636^†^	<0.0001

Next, we focused on cognitive impairment and developed a logistic regression model using factors that demonstrated significant differences in univariate analysis (Table 5). Remarkably, cognitive impairment was an independent predictor of worse 1-year mortality for the model (odds ratio 5.427, 95% confidence interval 1.385 -21.260, p = 0.0152). Moreover, we conducted sensitivity analysis for septic patients diagnosed with the Sepsis-3 definition only. In this analysis, we confirmed that cognitive impairment was robust and reliable predictor of death within 1-year due to sepsis, as defined by the Sepsis-3 definition.

**Table 5:** Odds ratio of cognitive impairment for predicting 1-year mortality. Logistic regression analysis was conducted to identify predictors of one-year mortality, utilizing cognitive impairment as well as factors that showed significant differences in the univariate analysis, such as male, presence of over 2 points of hematological component in SOFA score, and use of catecholamines. We calculated the odds ratio of cognitive impairment for 1-year mortality by univariate and multivariate analyses. Also, sensitivity analysis was conducted only in Sepsis-3-defined patients. Statistical significance in the univariate and multivariate analyses was defined as p< 0.05. N: number; OR: odds ratio; CI: confidence interval; SOFA: sequential organ failure assessment score

Methods	Population	N	OR	95%CI	P value (< 0.05)
Univariate	All sepsis cases	76	3.000	1.076 – 8.366	0.0358
Multivariate	All sepsis cases	76	5.427	1.385 – 21.260	0.0152
	Only Sepsis-3 cases	53	6.452	1.231 – 33.830	0.0274

**Figure 2:**
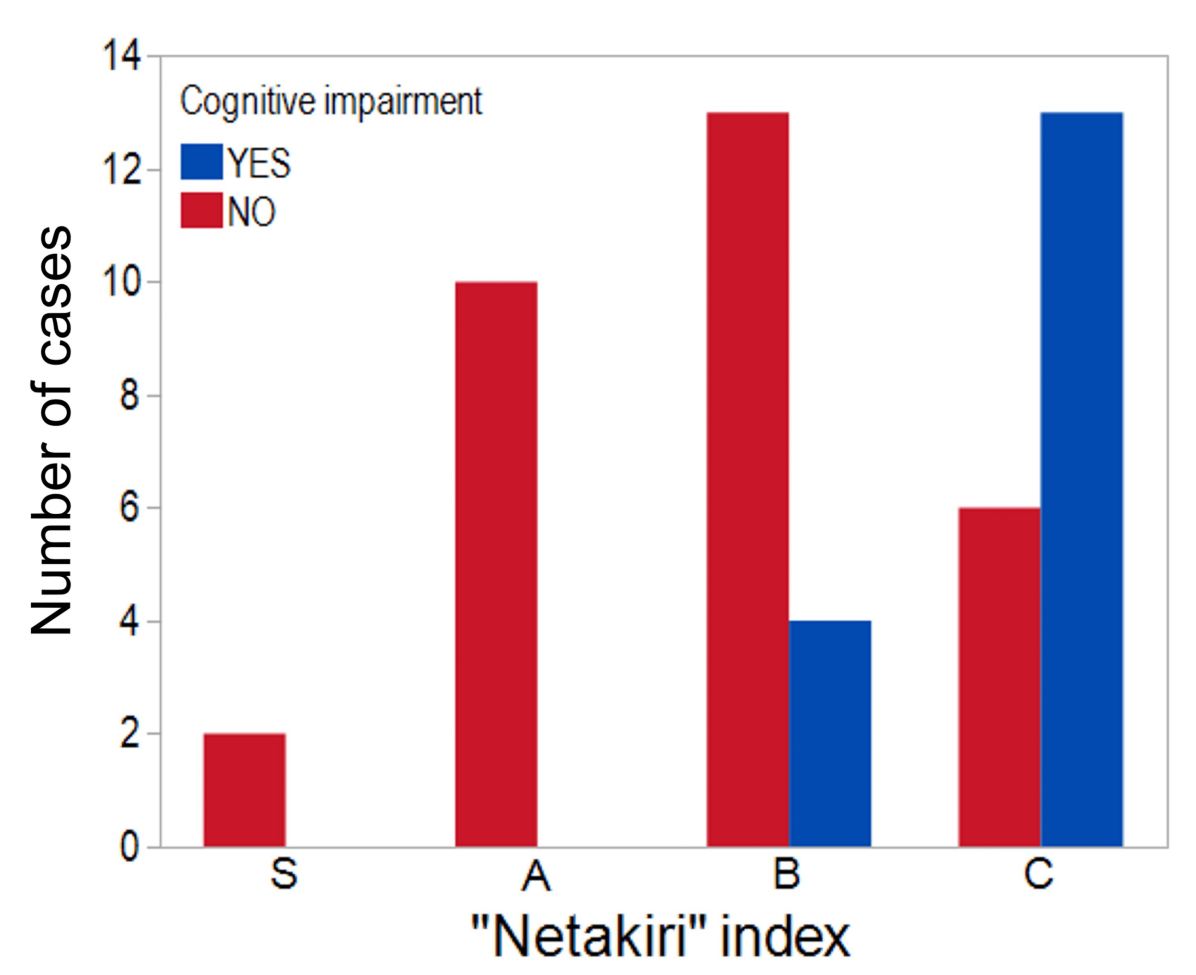
Pre-admission cognitive impairment and Netakiri index at discharge. All patients with cognitive impairment were bedridden at the time of discharge.

**Figure 3:**
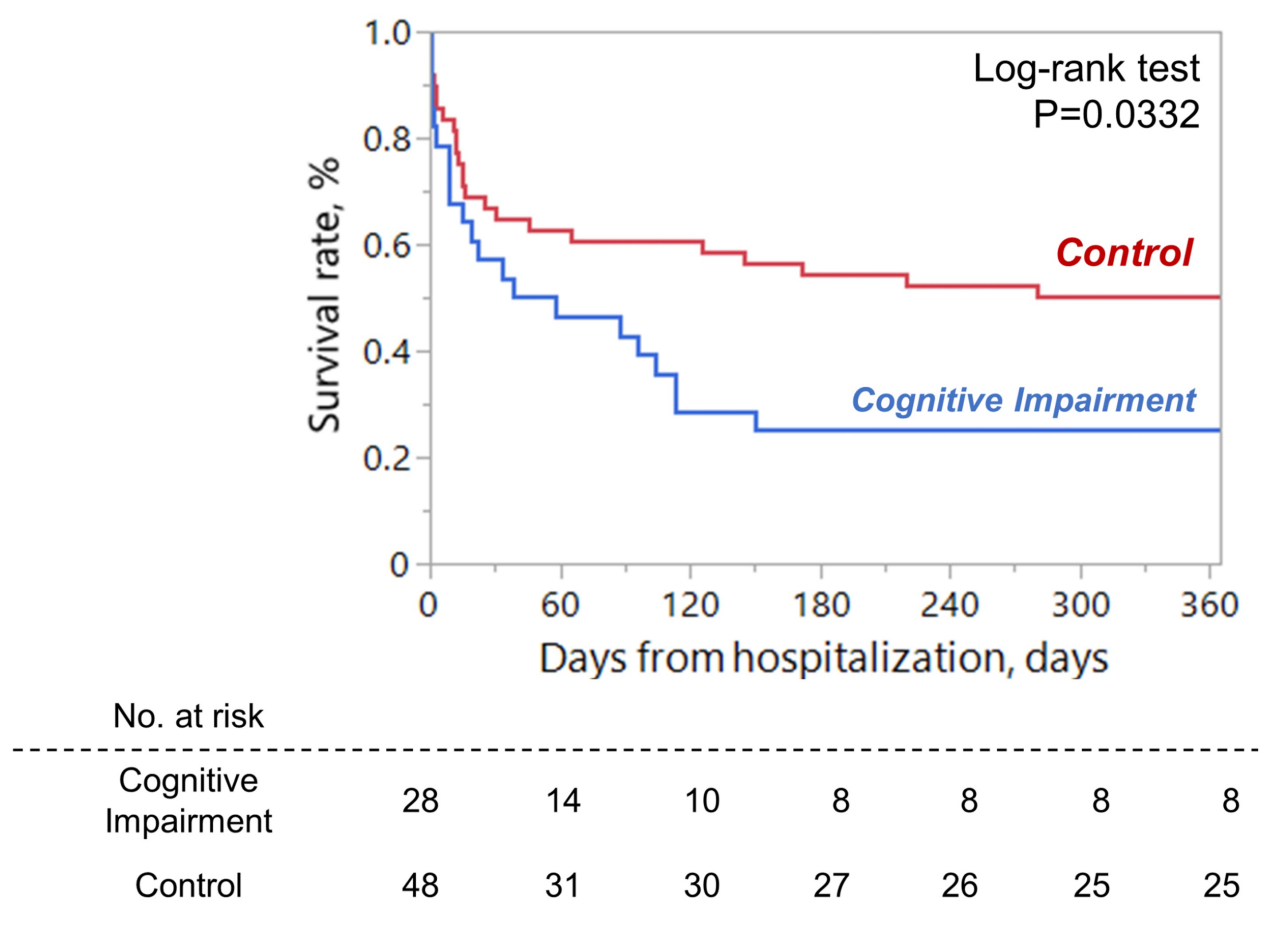
Cognitive impairment and 1-year mortality. Survival analyses with the log-rank test demonstrated that the cognitive impairment group had significantly lower survival. Statistical significance in the log-rank test was defined as p < 0.05.

Finally, we performed patient history and needs assessment regarding the presence or absence of cognitive impairment and clinical outcomes prior to admission. All patients with cognitive impairment had a bedridden index of B or C at discharge (Fig. 2). This means that all patients with cognitive impairment were discharged bedridden following treatment for sepsis. In addition, the log-rank test revealed a significantly lower survival rate in patients with cognitive impairment, as opposed to patients without (p=0.0332) (Fig. 3).

## Discussion

This retrospective study revealed that within a year of hospitalization and treatment for sepsis, 59% of patients died. Cognitive impairment was identified as an independent predictor of unfavorable outcomes one year after sepsis, regardless of the definition of sepsis.

In this study, 28 patients (36.8%) died during hospitalization, with a median hospital stay of just 12 days, despite receiving intensive sepsis care. Our patient cohort exhibited greater illness severity than the reported Japanese sepsis patient population, with a significantly higher rate of organ support and about twice the mortality rate [18]. It is noteworthy that 17 (35.4%) of the 48 patients who were discharged alive from our hospital died within a year of discharge. The high rate of long-term mortality after sepsis, as reported in the literature, persists for up to five years [5, 7].

When interpreting clinical outcomes of sepsis, it is crucial to consider the diverse patient characteristics associated with sepsis. In the present retrospective study, 56 (73.7%) of the 76 patients enrolled were elderly, and 60 (78.9%) were diagnosed with severe sepsis and septic shock, based on the Sepsis-2 definition. Additionally, there were fewer cases of respiratory infections as sources of sepsis, but more cases of urinary tract and soft tissue infections compared to recent reports from Japan [18, 19]. As we expected, sepsis severity was associated with death within 1 year; however, it was surprising that infection sources, bacterial profiles, and blood culture positivity were not related to long-term outcomes. This suggests that factors other than sepsis severity may significantly affect long-term prognosis following sepsis.

Post-intensive care syndrome (PICS) has recently received considerable attention and could be a prominent example of such factors. A single-center prospective study conducted in Japan found that 70% of patients experienced PICS three months after contracting sepsis [20]. The study identified Glasgow coma scale (GCS) on day 7 as an independent, reliable marker for PICS, rather than any variables related to sepsis. We confirmed the potential impact of various multidisciplinary treatments, including administration of catecholamines and steroids, as well as ventilatory management, on long-term prognosis. However, we did not evaluate PICS in our investigation, although we plan to explore further evidence in future studies.

The most significant finding of this study is that cognitive impairment prior to admission is an independent predictor of 1-year mortality from sepsis. The issue of cognitive impairment after sepsis is a significant concern for ICU patients, with rates of post-septic cognitive impairment ranging from 12.5% to 21% [11]. These acquired impairments are thought to be caused by pathophysiological changes such as blood-brain barrier dysregulation, neuroinflammation, neurotransmitter dysfunction, and neuronal loss, which are thought to be responsible for long-term cognitive impairments [21]. However, there has been little evidence available regarding the prevalence and impact of pre-existing cognitive impairment at the time of sepsis [10].

Upon admission to our emergency department, 36.8% of patients in this study had cognitive impairment. This rate is substantially higher than the 6.1% reported in a prospective cohort study [10]. Our findings suggest that this pre-existing vulnerability is a critical factor that can increase the severity of both short-term and long-term outcomes, potentially compounding the effects of the pathophysiological changes of sepsis. Therefore, our results strengthen the argument that both pre-existing and acquired impairments must be considered when evaluating the long-term prognosis of sepsis survivors.

In light of the above, early confirmation as well as post-sepsis follow-up may help to improve long-term outcomes of sepsis patients. At the very least, our data suggest that sepsis patients with cognitive impairment are highly prone to be bedridden or dead at hospital discharge. Early rehabilitation is one of the few effective options to prevent patients from becoming bedridden. Recent guidelines on rehabilitation for PICS indicate that cognitive rehabilitation can prevent the onset of PICS and can reduce symptoms such as spatial cognitive impairment [21]. Additionally, seamless cooperation between inpatient and outpatient rehabilitation programs should not be overlooked. In the present study, the median length of hospital stay was only 16 days, and sepsis patients stayed in smaller community hospitals, nursing facilities, or at home for more than 300 days. In the future, it is important for communities to consider consistent rehabilitation for sepsis patients after discharge from the hospital.

This study has several limitations. The small sample size, single-center status, and retrospective nature of this study limit the generalizability of our findings. It is also important to acknowledge that selection bias is an unavoidable limitation of a retrospective design. In addition, most enrolled sepsis patients were hospitalized based on the Sepsis-2 definition. Therefore, the results of this study may not be applicable to Sepsis-3 patients. All assessments of patient physical difficulties, cognitive impairment, emotional problems, and the Netakiri index were performed by nurses without officially certified assessment tools. This evaluation method may have caused inter-rater variability and misclassification bias. This also shows that there are limits to the objectivity of assessors.

The lack of a standardized sepsis treatment protocol at our hospital during the study period may have introduced variability in treatment approaches. This could potentially influence clinical outcomes, such as mortality and long-term disabilities, as patient management was dependent on individual physicians' discretion. Finally, unmeasured confounding factors such as socioeconomic status and baseline vulnerability cannot be ignored, as they have a potential impact on the interpretation of the results.

## Conclusions

This retrospective study found that 59% of sepsis patients died within 1 year of release from the hospital. Cognitive impairment was the most reliable independent predictor of poor outcomes. Sepsis patients with cognitive impairment are at high risk of being bedridden or dying by the time of hospital discharge. These findings suggest that incorporating a simple cognitive screening into admission protocols for sepsis patients may facilitate early identification of high-risk individuals and guide future research regarding the effectiveness of rehabilitation programs.

## References

[1] Fleischmann C, Scherag A, Adhikari NK (2016). Assessment of global incidence and mortality of hospital-treated sepsis. Current estimates and limitations. Am J Respir Crit Care Med.

[2] Dellinger RP, Carlet JM, Masur H (2004). Surviving Sepsis Campaign guidelines for management of severe sepsis and septic shock. Intensive Care Med.

[3] Angus DC, Carlet J (2003). Surviving intensive care: a report from the 2002 Brussels Roundtable. Intensive Care Med.

[4] Maley JH, Mikkelsen ME (2016). Short-term gains with long-term consequences: the evolving story of sepsis survivorship. Clin Chest Med.

[5] Shankar-Hari M, Ambler M, Mahalingasivam V, Jones A, Rowan K, Rubenfeld GD (2016). Evidence for a causal link between sepsis and long-term mortality: a systematic review of epidemiologic studies. Crit Care.

[6] Winters BD, Eberlein M, Leung J, Needham DM, Pronovost PJ, Sevransky JE (2010). Long-term mortality and quality of life in sepsis: a systematic review. Crit Care Med.

[7] Wang HE, Szychowski JM, Griffin R, Safford MM, Shapiro NI, Howard G (2014). Long-term mortality after community-acquired sepsis: a longitudinal population-based cohort study. BMJ Open.

[8] Prescott HC, Angus DC (2018). Enhancing recovery from sepsis: a review. JAMA.

[9] Guerra C, Hua M, Wunsch H (2015). Risk of a diagnosis of dementia for elderly medicare beneficiaries after intensive care. Anesthesiology.

[10] Iwashyna TJ, Ely EW, Smith DM, Langa KM (2010). Long-term cognitive impairment and functional disability among survivors of severe sepsis. JAMA.

[11] Calsavara AJ, Nobre V, Barichello T, Teixeira AL (2018). Post-sepsis cognitive impairment and associated risk factors: a systematic review. Aust Crit Care.

[12] Needham DM, Colantuoni E, Dinglas VD (2016). Rosuvastatin versus placebo for delirium in intensive care and subsequent cognitive impairment in patients with sepsis-associated acute respiratory distress syndrome: an ancillary study to a randomised controlled trial. Lancet Respir Med.

[13] Ehlenbach WJ, Gilmore-Bykovskyi A, Repplinger MD, Westergaard RP, Jacobs EA, Kind AJ, Smith M (2018). Sepsis survivors admitted to skilled nursing facilities: cognitive impairment, activities of daily living dependence, and survival. Crit Care Med.

[14] Iba T, Nisio MD, Levy JH, Kitamura N, Thachil J (2017). New criteria for sepsis-induced coagulopathy (SIC) following the revised sepsis definition: a retrospective analysis of a nationwide survey. BMJ Open.

[15] Gando S, Iba T, Eguchi Y (2006). A multicenter, prospective validation of disseminated intravascular coagulation diagnostic criteria for critically ill patients: comparing current criteria. Crit Care Med.

[16] Singer M, Deutschman CS, Seymour CW (2016). The third international consensus definitions for sepsis and septic shock (Sepsis-3). JAMA.

[17] Levy MM, Fink MP, Marshall JC (2003). 2001 SCCM/ESICM/ACCP/ATS/SIS International Sepsis Definitions Conference. Crit Care Med.

[18] Imaeda T, Oami T, Takahashi N, Saito D, Higashi A, Nakada TA (2023). Epidemiology of sepsis in a Japanese administrative database. Acute Med Surg.

[19] Wada T, Yamakawa K, Kabata D (2022). Age-related differences in the survival benefit of the administration of antithrombin, recombinant human thrombomodulin, or their combination in sepsis. Sci Rep.

[20] Inoue S, Nakanishi N, Sugiyama J (2022). Prevalence and long-term prognosis of post-intensive care syndrome after sepsis: a single-center prospective observational study. J Clin Med.

[21] Renner C, Jeitziner MM, Albert M (2023). Guideline on multimodal rehabilitation for patients with post-intensive care syndrome. Crit Care.

